# Design, Synthesis and DFT/DNP Modeling Study of New 2-Amino-5-arylazothiazole Derivatives as Potential Antibacterial Agents

**DOI:** 10.3390/molecules23020434

**Published:** 2018-02-15

**Authors:** Sraa Abu-Melha

**Affiliations:** Department of Chemistry, Faculty of Science of Girls, King Khaled University, Abha 62529, Saudi Arabia; sraa201313@yahoo.com or sabomlba@kku.edu.sa; Tel.: +966-504-757-797

**Keywords:** 4-aminoacetophenone, 2-amino-4-phenylthiazole, benzoyl chloride, modeling, DFT, antibacterial

## Abstract

A new series of 2-amino-5-arylazothiazole derivatives has been designed and synthesized in 61–78% yields and screened as potential antibacterial drug candidates against the Gram negative bacterium *Escherichia coli.* The geometry of the title compounds were being studied using the Material Studio package and semi-core pseudopods calculations (dspp) were performed with the double numerica basis sets plus polarization functional (DNP) to predict the properties of materials using the hybrid FT/B3LYP method. Modeling calculations, especially the (E_H_-E_L_) difference and the energetic parameters revealed that some of the title compounds may be promising tools for further research work and the activity is structure dependent.

## 1. Introduction

The thiazole ring is considered as a significant scaffold for the synthesis of several compounds with biological and pharmacological activities such as antibacterial [1], anti-inflammatory [2], antiprotozoal [3], antimalarial [4], anticancer [5] and anti-HIV [6] activities. Natural products containing the thiazole nucleus have been previously described as biologically active agents, for example vitamin B1 (thiamine) is an important precursor for the synthesis of acetylcholine, which improves the function of the nervous system [7], many common thiazole-containing antibiotics [8] are active against Gram-positive mycelial sporulating bacteria, largely of the genus *Streptomyces* [9]. Thiazole and its analogues have demonstrated efficacy in overcoming several CNS disorders in rodent as well as primate models [10]. A few drugs containing 2-aminothiazoles have been launched in the market, e.g., famotidine is used in the treatment of peptic ulcers and controls gastro-esophageal reflux [11], Abafungin is used in the treatment of dermatomycoses [12] and cefdinir is a semi-synthetic third generation broad spectrum cephalosporin antibiotic [13]. The aminothiazole moiety in cefdinir affects its pharmacodynamic properties in addition to its kinetics. Digestive tract iron (II) ions are believed to form chelate complexes with the thiazole nucleus, restricting the gastrointestinal absorption of cefdinir [14]. Sudoxicam [15] and meloxicam [16] behave as anti-inflammatory drugs and are used to treat arthritis, dysmenorrhea and fever.

## 2. Results and Discussion

### 2.1. Synthesis

**Scheme 1:**
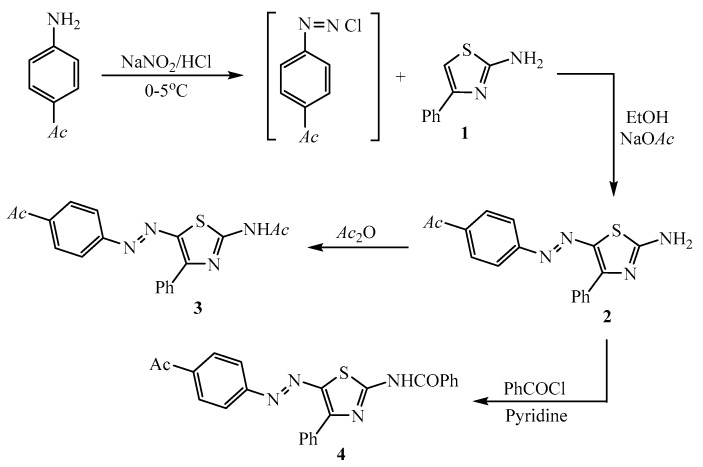
Acetylation and benzoylation of 5-(4-acetylphenylazo)-2-amino-4-phenylthiazole (**2**).

The reactivity of 2-amino-4-phenylthiazole (**1**) in the electrophilic diazo coupling reaction with diazotized *p*-aminoacetophenone has been investigated (Scheme 1); the reaction proceeded in ethyl alcohol and sodium acetate at the C5 position (active site) of the thiazole nucleus to afford the corresponding 5-(4-acetylphenylazo)-2-amino-4-phenylthiazole (**2**). Correct elemental and spectral analyses verified the chemical structure of this thiazole derivative. The IR absorptions at 3373 and 3297 cm^−1^ clearly characterized the NH_2_ function, while an absorption at 1669 cm^−1^ indicated the presence of a carbonyl group. The ^1^H-NMR spectrum showed a singlet at δ = 2.58 ppm for three protons (CH_3_), a multiplet in the δ = 7.50–8.24 ppm region for the aromatic protons, in addition to a singlet at δ = 8.62 ppm corresponding to the NH_2_ group.

Heating of 5-arylazo-2-aminothiazole derivative **2** with acetic anhydride on a water bath furnished 2-acetylamino-5-(4-acetylphenylazo)-4-phenylthiazole (**3**) in good yield. The chemical structure of **3** was secured by its correct elemental analysis and spectral data. The presence of an IR absorption at 3152 cm^−1^ clearly indicated an imino function (NH), while those at 1695 cm^−1^ and 1662 cm^−1^ correspond to the amide carbonyl function (NCOCH_3_) and the acetyl carbonyl function (ArCOCH_3_), respectively. The ^1^H-NMR spectrum exhibited two singlets at 2.23 and 2.60 ppm for the protons of two methyl groups (CH_3_), a multiplet in the region 7.49–8.25 ppm for the aromatic protons and a singlet at 12.69 ppm corresponding to the (NH) group proton.

*N*-Benzoylation of 5-arylazo-2-aminothiazole derivative **2** was achieved by stirring with benzoyl chloride in pyridine at room temperature to obtain 5-(4-acetylphenylazo)-2-benzoylamino-4-phenylthiazole (**4**). The structure of **4** was confirmed by elemental analysis and spectral data. In the IR spectrum, the presence of the (NH) group was indicted by the absorption at 3146 cm^−1^, while the two carbonyl functions (C=O) absorbed at 1732 and 1686 cm^−1^. The molecular ion peak at *m*/*z* = 426 with relative intensity 12% (mass spectrum) corresponded to the exact molecular weight of the formula C_24_H_18_N_4_O_2_S.

**Scheme 2:**
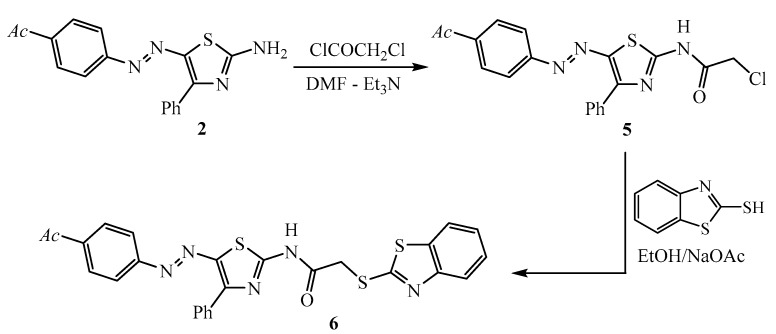
Chloroacetylation of 5-(4-acetylphenylazo)-2-amino-4-phenylthiazole (**2**).

In addition, chloroacetylation of 5-arylazo-2-aminothiazole derivative **2** was carried out by the reaction with chloroacetyl chloride in dimethylformamide containing a few drops of triethylamine to afford *N*-(5-(4-acetyl-phenylazo)-4-phenylthiazol-2-yl)-2-chloroacetamide (**5**) (Scheme 2). The chemical structure of **5** was identified from its correct spectroscopic data. The IR spectrum displayed an absorption at 3168 cm^−1^ for the (NH) group and two absorptions at 1700 and 1658 cm^−1^ for the two carbonyl functions (C=O). The ^1^H-NMR signals were two singlets at 2.55 and 4.15 ppm for the protons of the (CH_3_) and (CH_2_) groups and a multiplet in the region 7.01–8.11 ppm for the aromatic protons.

The reaction of chloroacetyl derivative **5** with 2-mercaptobenzothiazole was carried out by heating in ethyl alcohol containing sodium acetate to afford the expected sulfide product which was identified as 2-(benzothiazol-2-ylthio)-*N*-(4-phenyl-5-(4-acetylphenylazo)thiazol-2-yl)-acetamide (**6**). This proposed structure was supported by the correct spectroscopic data, for example, its IR spectrum exhibited an absorption at 3168 cm^−1^ corresponding to the (NH) group and two absorptions at 1699 and 1659 cm^−1^ for the two carbonyl functions (C=O). The molecular ion peak at *m*/*z* = 529 with relative intensity 14.61% (mass spectrum) clearly indicated the correct molecular weight of the formula C_26_H_19_N_5_O_2_S_3_.

**Scheme 3:**
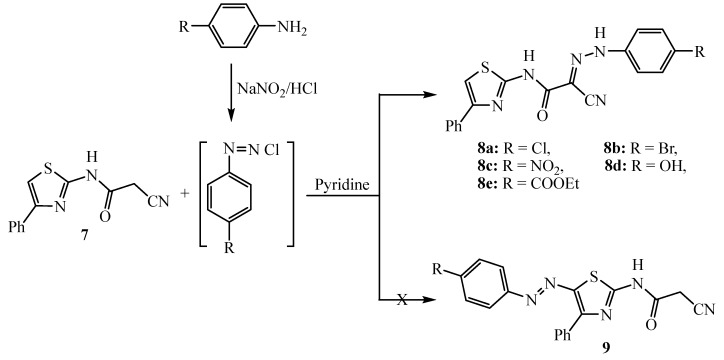
Diazocoupling of *N*-thiazolyl cyanoacetamide derivative **7** with aryl diazonium chlorides.

*N*-Thiazolyl cyanoacetamide derivative **7** [17] showed interesting reactivity toward a variety of chemical reagents. It is characterized by the presence of two active sites for electrophilic substitution reactions with aryldiazonium chlorides, so its reactivity towards such diazonium species was studied. The methylene function proved to be more reactive towards diazo-coupling reaction with aryldiazonium chlorides than the thiazole C-5. Thus, diazo-coupling reaction of compound **7** with diazotized substituted anilines (namely: 4-chloroaniline, 4-bromoaniline, 4-nitroaniline, 4-amino-phenol and ethyl 4-aminobenzoate) proceeded in pyridine at 0–5 °C to furnish the corresponding *N*-(4-phenylthiazol-2-yl)-2-arylhydrazono-2-cyanoacetamide derivatives **8a**–**e** and failed to give the alternative 5-arylazothiazoles **9** (Scheme 3). The chemical structure of thiazoles **8a**–**e** was established from their elemental analysis and spectral data. In the IR spectrum of **8a**, for example, the presence of absorptions at 3223 and 3186 cm^−1^ indicated (NH) functions, along with bands at 2218 cm^−1^ for the nitrile function (C≡N) and 1657 cm^−1^ for the carbonyl group (C=O). The ^1^H-NMR spectrum of **8b** displayed a multiplet in the 7.35–8.05 ppm region for the aromatic and thiazole-C_5_ protons and two singlets at 12.05 and 12.65 ppm corresponding to the protons of two (NH) functions.

### 2.2. Computational Studies

Cluster calculations were carried out using DMOL^3^ program [18] in the Materials Studio package [19] designed for the realization of large scale density functional theory (DFT) calculations with double numerical basis sets plus polarization functional (DNP) that proved to be of high accuracy than 6–31 G Gaussian basis sets of an equivalent size [20,21,22]. The geometric optimization is performed with none symmetry restriction.

#### 2.2.1. Geometry Optimization with the DFT Method

**Figure 1:**
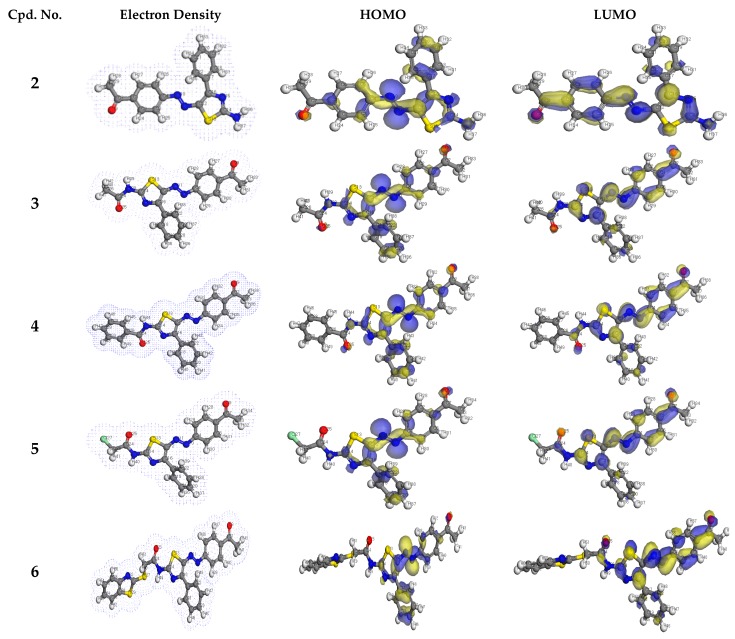
The electron density, the HOMO and LUMO frontier orbitals of 5-(4-acetylphenylazo) thiazoles **2**, **3**, **4**, **5** and **6**.

**Figure 2:**
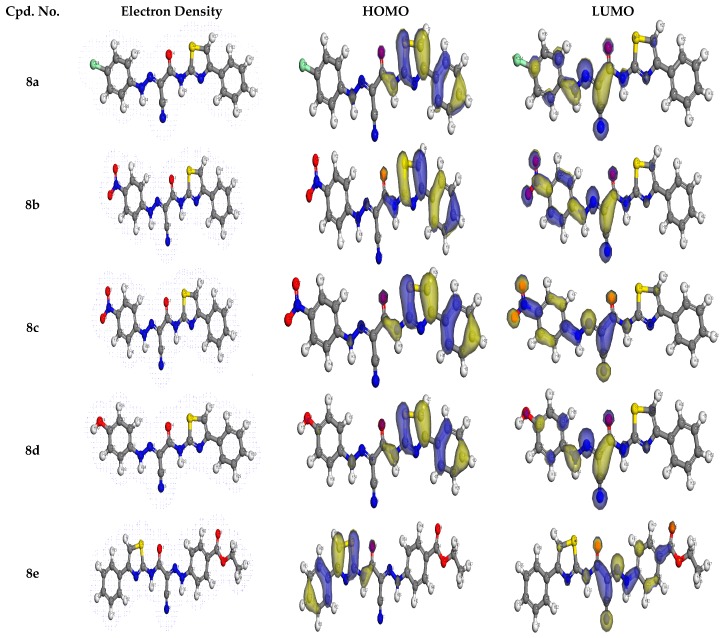
The electron density, the HOMO and LUMO frontier orbitals of *N*-(thiazol-2-yl)-2-arylhydrazono-2-cyanoacetamides **8a**–**e**.

Figure 1 and Figure 2 present the molecular structures along with the atom numbering of the thiazole derivatives (compounds **2**, **3**, **4**, **5**, **6** and **8a**–**e**) and the data, including the bond lengths and bond angles, are recorded in the Supplementary Material (Tables S1–S20). There are clear changes in the bond lengths and angles upon the formation of a new derivatives, depending on the nature and the position on the arylazothiazole nucleus where the entering molecule is added.

#### 2.2.2. Global Reactivity Descriptors

In quantum chemical calculations, it is very important to determine the energies (*E*_HOMO_ and *E*_LUMO_) of the HOMO (π donor) which is the orbital that primarily acts as an electron donor and LUMO (π acceptor) that is the orbital that largely act as the electron acceptor, respectively. These molecular orbitals are also called the frontier molecular orbitals (FMOs). The values, listed in Table 1, indicates that E_HOMO_ and E_LUMO_ and their neighboring orbitals are negative revealing the stability of the prepared thiazole derivative molecules [23]. On the basis of the fact that, the overlap between the frontier molecular orbitals (FMOs) is considered to be controlling factor in many reactions. Thus, orbitals of the thiazole derivative with the largest value of molecular orbital coefficients may be considered as the sites of electron donation. Figure 1 and Figure 2 illustrate that the HOMO level is mostly localized on the protonated S(13), C(15), N(9), N(7) and C(10) atoms in the thiazole moiety, suggesting they will be the most preferable sites for nucleophilic attack for the entering group. Also, the energy gap (*E*_HOMO_-*E*_LUMO_) that is an important stability index helps to characterize the chemical reactivity and kinetic stability of the molecule [23]. It is a fact that, the smaller the gap that occurs due to the groups that enter into conjugation is, the more polarized the molecule is and in turn, the molecule is a soft one and easily offer electrons to an acceptor [24]. This in turn affects the biological activity of the molecule. To ascertain the biological activity of the thiazole derivatives, some additional parameters such as the electronegativity (*χ*), chemical potential (*µ*), global hardness (*η*), global softness (*S*) and global electrophilicity index (*ω*) [25] are evaluated by the following equations and recorded in Table 1:

*χ* = −1/2 (*E*_LUMO_ + *E*_HOMO_) (1)

*µ* = −*χ* =1/2 (*E*_LUMO_ + *E*_HOMO_) (2)

*η* = 1/2 (*E*_LUMO_ − *E*_HOMO_) (3)

*S* = 1/2 *η* (4)

*ω = µ*^2^/2 *η* (5)

The inverse value of the global hardness is designed as the softness *σ* as follows:

*σ* = 1/*η* (6)

The electrophilicity index (*ω*) comprises the most important quantum chemical descriptors in providing a good idea about the toxicity of different pollutants so the quantifies the biological activity of drug receptor interactions as it measures the stabilization in energy when the system acquires an additional electronic charge from the surrounding environment [26]. Thus, a glance at the data listed in Table 1 indicates that compounds can be arranged according to their energy gaps (*E*_HOMO_*-E*_LUMO_) as: **8a** > **8d** > **8e** > **2** > **4** = **5** > **3** > **6** > **8c** > **8b**. This is consistent with the fact that compound **8b** has the smallest *E*_LUMO_-*E*_HOMO_ (−1.338 e.V.) (Table 1) and the highest electrophilicity index (*ω* = 14.987) among the derivatives under study. Consequently, the derivative **8b** will be the more soft, polarized, and more reactive the others, as it easily offer electrons to an acceptor indicating that charge transfer easily occurs in it which in turn makes it a promising biologically active compound. Furthermore, general the calculations of the binding energy revealed an increase of the value of the calculated binding energy of the new thiazole compounds compared to that of the start, indicating the higher stability of the formed compounds [26].

### 2.3. Antibacterial Activity

The activity of these synthesized thiazole derivatives was individually evaluated against the Gram-negative bacterium *Escherichia coli* ATCC 25922 by the agar diffusion method. A solution of the thiazole derivative in DMSO was prepared separately with a concentration of 1 mg/mL. Paper discs of Whatman filter paper were cut to a standard size (5 cm) and sterilized in an autoclave. The paper discs, soaked in the desired concentration of the complex solution, were placed aseptically in Petri dishes containing nutrient agar media (agar 20 g + beef extract 3 g + peptone 5 g) seeded with *E. coli*. The Petri dishes were incubated at 36 °C and the inhibition zones were measured after 24 h of incubation. Each treatment was replicated three times and the average value is recorded. The antibacterial activity of ampicillin (a standard antibiotic) was also investigated under the same conditions as above [27]. The % activity index for the compound was calculated by the formula:

% Activity Index = Zone of inhibition by test compound (diameter)/Zone of inhibition by standard (diameter) × 100 (7)

The results are given in Table 2. Ampicillin (inhibition zone 24 nm) was used as standard antibacterial reference. Thiazole compound **6** exhibited the highest potency against the tested organism compared to the reference drug. It inhibited the growth of *Escherichia coli* with an inhibition zone of 19 mm, respectively. The inhibition activity of the title compounds follows the order: **6** > **8b** > **8a** = **8d** > **5** > **8c** > **3** > **2**. The variation in the effectiveness of different compounds against a bacterial organism depends on either the impermeability of the cells of the microbes or on differences in the ribosomes of microbial cells [28]. It may be concluded that the antimicrobial activity of the compounds is related to cell wall structure of the bacterium as well as the structure of the thiazole itself. It is possible because the cell wall is essential to the survival of bacteria and some antibiotics are able to kill bacteria by inhibiting a step in the synthesis of peptidoglycan. Gram-positive bacteria possess a thick cell wall containing many layers of peptidoglycan and teichoic acids, but in contrast, Gram negative bacteria such as *E. coli* that is utilized in our present work has a relatively thin cell wall consisting of a few layers of peptidoglycan surround by a second lipid membrane containing lipopolysaccharides and lipoproteins. These differences in cell wall structure can produce differences in antibacterial susceptibility and some antibiotics can kill only Gram-positive bacteria and is ineffective against Gram-negative pathogens [29].

On the other hand it is obvious that compound **6** exhibited potent inhibition activity owing to its characteristic skeleton that containing three S atoms that confer it softness, five donating N atoms and three planar phenyl groups in excess when compared with the other derivatives which makes it more polarizable, soft and reactive than other derivatives, thus facilitating its permeability through the cell membrane of the bacterium. Thus it is able to diffuse through the cell wall of the bacterium and hence capable to interfere with its biological activity or it can diffuse and inactivate the bacterium by some unknown cellular mechanism. The importance of such work lies in the possibility that the new compounds might be more effective drugs against bacteria for which a thorough investigation regarding the structure–activity relationship, toxicity and in their biological effects which could be helpful in designing more potent antibacterial agents for therapeutic use.

## 3. Materials and Methods

### 3.1. General Methods

All melting points were measured on Gallenkamp electric melting point apparatus (capillary method, Gallenkamp Co., London, UK). Infrared spectra were determined on a Mattson 5000 FT-IR spectrometer (Shimadzu Co., Kyoto, Japan, not all frequencies are reported) using KBr discs. The ^1^H-NMR and ^13^C-NMR spectra were acquired using a WP 300 spectrometer (Bruker Co., Billerica, MA, USA) at 300 MHz (^1^H) or 75.5 MHz (^13^C; in broad band mode). The NMR spectra of the thiazole products **4** and **6** are not provided due to their limited solubility in common NMR solvents. The mass spectra were recorded using a Qp-2010 mass spectrometer (Shimadzu, Tokyo, Japan) at 70 eV (EI mode). Elemental analyses (C, H and N) were determined on Perkin-Elmer 2400 analyzer (Perkin-Elmer, Waltham, MA, USA).

### 3.2. Synthesis of 5-(4-Acetylphenylazo)-4-phenyl-2-substituted thiazoles ***2**–**6***

*5-(4-Acetylphenylazo)-2-amino-4-phenylthiazole* (**2**). A well stirred suspension of 4-aminoacetophenone (20 mmol, 2.7 g) in concentrated hydrochloric acid (6.0 mL) was cooled to 0–5 °C and then diazotized with a solution of NaNO_2_ (1.4 g in 15 mL H_2_O). The freshly prepared diazonium solution was added dropwise to a stirred cold solution of the 2-amino-4-phenylthiazole (**1**, 20 mmol, 3.54 g) and sodium acetate (6 g) in ethyl alcohol (20 mL). The reaction mixture was stirred at 0–5 °C for an hour to achieve complete diazo-coupling reaction. The solid product was collected by filtration, washed with water and recrystallized by heating in ethyl alcohol. Dark red crystals, yield = 78%, m.p. = 254–256 °C. IR ($\bar{\nu }$/cm^−1^): 3373, 3297 (NH_2_), 1669 (C=O), 1647 (C=N). ^1^H-NMR (DMSO-d_6_): δ 2.58 (s, 3H, COCH_3_), 7.50–8.24 (m, 9H, Ar-H), 8.62 (s, 2H, NH_2_). ^13^C-NMR (DMSO-d_6_): δ 26.82, 122.46 (2C), 126.24, 127.68 (2C), 128.81 (2C), 130.05 (2C), 134.32, 136.22, 137.76, 140.54, 150.36, 165.26, 197.06. Analysis for C_17_H_14_N_4_OS (322.39): Calcd: C, 63.34; H, 4.38; N, 17.38%. Found: C, 63.43; H, 4.32; N, 17.27%.

*2-Acetylamino-5-(4-acetylphenylazo)-4-phenylthiazole* (**3**). 5-(4-Acetylphenylazo)-2-amino-4-phenyl-thiazole (**2**, 2 mmol, 0.64 g) was heated in acetic anhydride (5 mL) at 70–75 °C for 2 h. The solid product was separated by recrystallization from 20 mL ethyl alcohol to give the acetyl compound **3**. Dark red crystals, yield = 74%, m.p. = 285–287 °C. IR ($\bar{\nu }$/cm^−1^): 3152 (NH), 1695 (C=O), 1662 (C=O). ^1^H-NMR (DMSO-*d*_6_): δ/ppm = 2.23 (s, 3H, CH_3_), 2.60 (s, 3H, CH_3_), 7.49–8.25 (m, 9H, Ar-H), 12.69 (s, 1H, NH). ^13^C-NMR (DMSO-*d*_6_): δ 22.54, 26.68, 122.84 (2C), 126.41, 128.62 (2C), 129.38 (2C), 129.94 (2C), 134.26, 137.56, 141.65, 148.48, 151.51, 160.14, 168.52, 197.18. Analysis for C_19_H_16_N_4_O_2_S (364.42): Calcd: C, 62.62; H, 4.43; N, 15.37%. Found: C, 62.70; H, 4.47; N, 15.43%.

*5-(4-Acetylphenylazo)-2-benzoylamino-4-phenylthiazole* (**4**). To solution of compound **2** (2 mmol, 0.64 g) in pyridine (20 mL), benzoyl chloride (2 mmol, 0.44 mL) was added and stirred for 2 h at room temperature. Then, a solution of sodium acetate (1 g in 10 mL cooled H_2_O) was added to the reaction mixture and the precipitated product was filtered off and recrystallized by heating in ethyl alcohol. Reddish brown powder, yield = 70%, m.p. = 274–275 °C. IR ($\bar{\nu }$/cm^−1^): 3146 (NH), 1732 (C=O), 1686 (C=O). MS (EI): *m*/*z* (%) = 426 (molecular ion, 12), 268 (25.38), 120 (71.67), 79 (37.95), 68 (40.17), 56 (base peak, 100), 45 (57.31). Analysis for C_24_H_18_N_4_O_2_S (426.49): Calcd: C, 67.59; H, 4.25; N, 13.14%. Found: C, 67.68; H, 4.19; N, 13.05%.

*N-(5-(4-Acetylphenylazo)-4-phenylthiazol-2-yl)-2-chloroacetamide* (**5**). 2-Chloroacetyl chloride (10 mmol, 0.8 mL) was added to a suspension of 2-amino-5-arylazothiazole **2** (5 mmol, 1.62 g) in dimethyl formamide (30 mL) containing five drops of triethylamine. The reaction mixture was stirred at 25–30 °C for 4 h and then poured into ice-cold water. The solid that formed was picked up by filtration, dried and recrystallized from ethyl alcohol. Orange crystals, yield = 62%, m.p. = 228–230 °C. IR ($\bar{\nu }$/cm^−1^): 3168 (NH), 1700 (C=O), 1658 (C=O). ^1^H-NMR (DMSO-*d*_6_): δ 2.55 (s, 3H, CH_3_), 4.15 (s, 2H, CH_2_), 7.01–8.11 (m, 10H, Ar-H). ^13^C-NMR (DMSO-*d*_6_): δ 26.62, 42.34, 122.71 (2C), 126.70, 128.88 (2C), 130.09 (2C), 130.63 (2C), 135.13, 136.24, 141.21, 147.52, 150.70, 158.75, 166.68, 197.02. Analysis for C_19_H_15_ClN_4_O_2_S (398.87): Calcd: C, 57.21; H, 3.79; N, 14.05%. Found: C, 57.08; H, 3.83; N, 14.12%.

*2-(Benzo[d]thiazol-2-ylthio)-N-(4-phenyl-5-(4-acetyl-phenylazo)-thiazol-2-yl)-acetamide* (**6**). A mixture of **5** (1 mmol, 0.40 g), 2-mercaptobenzothiazole (1 mmol, 0.17 g) and sodium acetate (0.4 g) was refluxed in 20 mL ethanol for 4 h. The reaction mixture was allowed to cool at room temperature and then diluted with water (5 mL). The resulting solid was collected by filtration, dried and recrystallized from ethyl alcohol. Red powder, yield = 77%, m.p. = 260–261 °C. IR ($\bar{\nu }$/cm^−1^): 3168 (NH), 1699 (C=O), 1659 (C=O). MS (EI): *m*/*z* (%) = 529 (molecular ion, 14.61), 400 (16.33), 321 (base peak, 100), 165 (71.22), 133 (36.45), 84 (34.90). Analysis for C_26_H_19_N_5_O_2_S_3_ (529.65): Calcd: C, 58.96; H, 3.62; N, 13.22%. Found: C, 58.90; H, 3.61; N, 13.19%.

### 3.3. Synthesis of N-(4-Phenylthiazol-2-yl)-2-arylhydrazono-2-cyano-acetamide derivatives ***8a**–**e***

A well stirred suspension of the appropriate *para*-substituted aniline derivative (5 mmol) in concentrated HCl (1.5 mL) and water (3 mL) was cooled in an ice-bath at 0–5 °C and diazotized with a solution of NaNO_2_ (0.35 g) in H_2_O (10 mL). Then, the freshly cold diazonium solution was added dropwise to a well-stirred cold solution of compound **7** (5 mmol, 1.22 g) in pyridine (20 mL). The reaction mixture was stirred for 2 h to achieve a complete diazo-coupling reaction. The solid product was collected by filtration, washed with cold water, and recrystallized by heating in a mixture of ethyl alcohol/DMF (2:1).

*N-(4-Chlorophenyl)-2-oxo-2-((4-phenylthiazol-2-yl)amino)aceto-hydrazonoyl cyanide* (**8a**). Orange crystals, yield = 78%, m.p. = 210–211 °C. IR ($\bar{\nu }$/cm^−1^): 3223 (NH), 3186 (NH), 2218 (C≡N), 1657 (C=O). ^1^H-NMR (DMSO-*d*_6_): δ 7.25–8.00 (m, 10H, Ar-H and thiazole-H5), 12.15 (s, 1H, NH), 12.72 (s, 1H, NH). ^13^C-NMR (DMSO-*d*_6_): δ 104.41, 108.27, 109.63, 116.83 (2C), 126.38 (2C), 127.67, 129.31 (2C), 129.80, 130.51 (2C), 131.18, 136.73, 138.54, 142.84, 162.37. Analysis for C_18_H_12_ClN_5_OS (381.84): Calcd: C, 56.62; H, 3.17; N, 18.34%. Found: C, 56.87; H, 3.21; N, 18.45%.

*N-(4-Bromophenyl)-2-oxo-2-((4-phenylthiazol-2-yl)amino)aceto-hydrazonoyl cyanide* (**8b**). Brown crystals, yield = 70%, m.p. = 245–246 °C. IR ($\bar{\nu }$/cm^−1^): 3297 (NH), 2221 (C≡N), 1664 (C=O). ^1^H-NMR (DMSO-*d*_6_): δ 7.35–8.05 (m, 10H, Ar-H and thiazole-H5), 12.05 (s, 1H, NH), 12.65 (s, 1H, NH). ^13^C-NMR (DMSO-*d*_6_): δ 104.54, 108.38, 110.48, 116.57, 117.41 (2C), 127.54 (2C), 129.06 (2C), 129.75, 131.22, 132.51 (2C), 136.26, 138.83, 143.14, 162.81. Analysis for C_18_H_12_BrN_5_OS (426.29): Calcd: C, 50.72; H, 2.84; N, 16.43%. Found: C, 50.63; H, 2.89; N, 16.40%.

*N-(4-Nitrophenyl)-2-oxo-2-((4-phenylthiazol-2-yl)amino)acetohydrazonoyl cyanide* (**8c**). Brown powder, yield = 66%, m.p. = 248–250 °C. IR ($\bar{\nu }$/cm^−1^): 3419 (NH), 2230 (C≡N), 1648 (C=O). ^1^H-NMR (DMSO-*d*_6_): δ 7.20–8.20 (m, 10H, Ar-H and thiazole-H5), 12.43 (s, 1H, NH), 12.92 (s, 1H, NH). ^13^C-NMR (DMSO-*d*_6_): δ 106.07, 110.16, 112.33, 115.27 (2C), 124.58 (2C), 127.16 (2C), 129.47 (2C), 130.38, 134.46, 138.37, 141.63, 146.21, 148.33, 163.14. Analysis for C_18_H_12_N_6_O_3_S (392.39): Calcd: C, 55.10; H, 3.08; N, 21.42%. Found: C, 55.23; H, 3.04; N, 21.35%.

*N-(4-Hydroxyphenyl)-2-oxo-2-((4-phenylthiazol-2-yl)amino)aceto-hydrazonoyl cyanide* (**8d**). Brown crystals, yield = 61%, m.p. = 185–188 °C. IR ($\bar{\nu }$/cm^−1^): 3210 (OH), 3120 (NH), 2218 (C≡N), 1662 (C=O). ^1^H-NMR (DMSO-*d*_6_): δ 6.84–8.10 (m, 10H, Ar-H and thiazole-H5), 9.50 (s, 1H, OH), 11.85 (s, 1H, NH), 12.71 (s, 1H, NH). ^13^C-NMR (DMSO-*d*_6_): δ 105.81, 107.57, 110.36, 116.47 (2C), 118.84 (2C), 127.03 (2C), 129.22, 129.86 (2C), 132.26, 134.48, 138.27, 144.58, 147.84, 162.74. Analysis for C_18_H_13_N_5_O_2_S (363.40): Calcd: C, 59.49; H, 3.61; N, 19.27%. Found: C, 59.62; H, 3.69; N, 19.41%.

*N-(4-Ethoxycarbonylphenyl)-2-oxo-2-((4-phenylthiazol-2-yl)amino)aceto-hydrazonoyl cyanide* (**8e**). Red crystals, yield = 74%, m.p. = 255–257 °C. IR ($\bar{\nu }$/cm^−1^): 3394 (NH), 3291 (NH), 2223 (C≡N), 1702 (C=O), 1642 (C=O). ^1^H-NMR (DMSO-*d*_6_): δ 1.34 (t, 3H, CH_3_), 4.33 (q, 2H, OCH_2_), 7.50–8.24 (m, 10H, Ar-H), 8.67 (s, 1H, thiazole-H5). ^13^C-NMR (DMSO-*d*_6_): δ 14.24, 60.64, 103.67, 108.71, 109.48, 115.74 (2C), 121.47, 127.72 (2C), 128.43, 129.49 (2C), 130.78 (2C), 133.24, 145.79, 147.71, 148.33, 162.34, 166.42. Analysis for C_21_H_17_N_5_O_3_S (419.46): Calcd: C, 60.13; H, 4.09; N, 16.70%. Found: C, 60.25; H, 4.14; N, 16.79%.

## 4. Conclusions

The research reports on the synthesis of a new 5-(4-acetylphenylazo)-2-amino-4-phenylthiazole and its *N*-acetyl, *N*-benzoyl and *N*-chloroacetyl derivatives. The reaction of 2-*N*-cyanoacetamido-4-phenylthiazole with various aryl diazonium chlorides furnished the corresponding *N*-(4-phenylthiazol-2-yl)-2-arylhydrazono-2-cyanoacetamide derivatives via diazo-coupling at the methylene function rather than at the C5 position of the thiazole. The biological activity of these synthesized thiazoles were evaluated against the Gram negative bacterium *Escherichia coli.* The geometry of the title compounds was studied using the Material Studio package and semi-core pseudopods calculations (dspp) were performed with the double numerica basis sets plus polarization functional (DNP) to predict properties of materials using by the hybrid FT/B3LYP method.

## Figures and Tables

**Table 1:** Calculated EHOMO, ELUMO, electronegativity (*χ*), chemical potential (*μ*), global hardness (*η*), global softness (*S*), global electrophilicity index (*ω*) and softness (*σ*) for thiazole derivatives complexes.

Cpd. No.	*E*_HOMO_	*E*_LUOM_	*E*_HOMO_-*E*_LUOM_	*χ*	*μ*	*η*	*S*	*ω*	*σ*	Binding Energy
**2**	−4.718	−3.128	−1.590	3.923	−3.923	0.795	0.629	9.679	1.258	−4292.325
**3**	−4.493	−2.916	−1.577	3.704	−3.704	0.788	0.634	8.702	1.268	−4865.082
**4**	−4.872	−3.287	−1.585	4.079	−4.079	0.792	0.631	10.499	1.262	−5828.195
**5**	−4.938	−3.353	−1.585	4.145	−4.145	0.792	0.631	10.842	1.262	−4844.288
**6**	−4.885	−3.317	−1.568	4.101	−4.101	0.784	0.638	10.726	1.275	−6461.612
**8a**	−4.684	−2.857	−1.827	3.771	−3.771	0.913	0.547	7.781	1.095	−4488.996
**8b**	−5.147	−3.809	−1.338	4.478	−4.478	0.669	0.747	14.987	1.495	−4724.220
**8c**	−5.127	−3.78	−1.347	4.453	−4.453	0.6735	0.742	14.724	1.485	−4725.903
**8d**	−4.802	−3.047	−1.755	3.924	−3.924	0.8775	0.569	8.7758	1.139	−4618.547
**8e**	−5.392	−3.654	−1.738	4.523	−4.523	0.869	0.575	11.771	1.151	−5491.744

**Table 2:** Antibacterial activities expressed as inhibition diameter zones in millimeters (mm) of the tested compounds against *Escherichia coli*.

Compound Number	*E. coli* (mg/mL)	
	Diameter of Inhibition Zone (in mm)	% Activity Index
**2**	8	33.3
**3**	12	50.0
**4**	NA	----
**5**	15	62.5
**6**	19	79.2
**8a**	16	66.7
**8b**	18	75.0
**8c**	14	58.3
**8d**	16	66.7
**8e**	NA	----
Ampicillin	24	100

NA = No Activity.

## Supplementary Materials

Supplementary materials are available online.
