# Supplementary material for: Design, Synthesis and DFT/DNP Modeling Study of New 2-Amino-5-arylazothiazole Derivatives as Potential Antibacterial Agents
Source: Molecules. 2018 Feb 15;23(2):434. doi: 10.3390/molecules23020434 (PMC6017174; doi:10.3390/molecules23020434)
Supplement: Supplementary file 1 [file molecules-23-00434-s001.pdf]

Table S1: Selected bond lengths (Å) of compound 2 using DFT-method from DMOL3 calculations.

Bond	Length(Å)	Bond	Length(Å)	Bond	Length(Å)
N(23)-H(37)	1.015	C(16)-C(17)	1.482	C(7)-C(8)	1.526
N(23)-H(36)	1.017	N(15)-C(16)	1.374	C(6)-H(27)	1.09
C(22)-H(35)	1.088	C(14)-N(23)	1.378	C(5)-H(26)	1.09
C(21)-H(34)	1.091	C(14)-N(15)	1.328	C(5)-C(6)	1.394
C(21)-C(22)	1.396	S(13)-C(14)	1.735	C(4)-N(10)	1.41
C(20)-H(33)	1.092	C(16)-C(12)	1.420	C(4)-C(5)	1.414
C(20)-C(21)	1.403	C(12)-S(13)	1.773	C(3)-H(25)	1.089
C(19)-H(32)	1.092	N(11)-C(12)	1.375	C(3)-C(4)	1.419
C(19)-C(20)	1.401	N(10)-N(11)	1.292	C(2)-H(24)	1.09
C(18)-H(31)	1.089	C(8)-H(30)	1.096	C(2)-C(3)	1.39
C(18)-C(19)	1.399	C(8)-H(29)	1.104	C(1)-C(7)	1.5
C(22)-C(17)	1.411	C(8)-H(28)	1.100	C(6)-C(1)	1.412
C(17)-C(18)	1.410	C(7)-O(9)	1.241	C(1)-C(2)	1.418

Table S2: Selected bond angles (°) of compound 2 using DFT-method from DMOL3 calculations.

Angle	Degree(°)	Angle	Degree(°)
H(37)-N(23)-H(36)	114.97	S(13)-C(12)-N(11)	112.908
H(37)-N(23)-C(14)	117.659	C(12)-N(11)-N(10)	116.17
H(36)-N(23)-C(14)	113.569	N(11)-N(10)-C(4)	115.149
H(35)-C(22)-C(21)	119.587	H(30)-C(8)-H(29)	109.322
H(35)-C(22)-C(17)	119.961	H(30)-C(8)-H(28)	109.642
C(21)-C(22)-C(17)	120.426	H(30)-C(8)-C(7)	108.758
H(34)-C(21)-C(22)	119.348	H(29)-C(8)-H(28)	107.146
H(34)-C(21)-C(20)	120.208	H(29)-C(8)-C(7)	110.643
C(22)-C(21)-C(20)	120.421	H(28)-C(8)-C(7)	111.302
H(33)-C(20)-C(21)	119.897	O(9)-C(7)-C(8)	120.151
H(33)-C(20)-C(19)	120.476	O(9)-C(7)-C(1)	121.582
C(21)-C(20)-C(19)	119.623	C(8)-C(7)-C(1)	118.265
H(32)-C(19)-C(20)	120.319	H(27)-C(6)-C(5)	118.854
H(32)-C(19)-C(18)	119.623	H(27)-C(6)-C(1)	120.413
C(20)-C(19)-C(18)	120.057	C(5)-C(6)-C(1)	120.72
H(31)-C(18)-C(19)	120.432	H(26)-C(5)-C(6)	121.488
H(31)-C(18)-C(17)	118.836	H(26)-C(5)-C(4)	117.97
C(19)-C(18)-C(17)	120.726	C(6)-C(5)-C(4)	120.512
C(22)-C(17)-C(18)	118.711	N(10)-C(4)-C(5)	114.396
C(22)-C(17)-C(16)	122.371	N(10)-C(4)-C(3)	126.42
C(18)-C(17)-C(16)	118.836	C(5)-C(4)-C(3)	119.149
C(17)-C(16)-N(15)	116.853	H(25)-C(3)-C(4)	118.886
C(17)-C(16)-C(12)	128.366	H(25)-C(3)-C(2)	121.227
N(15)-C(16)-C(12)	114.780	C(4)-C(3)-C(2)	119.887
C(16)-N(15)-C(14)	111.422	H(24)-C(2)-C(3)	120.943
N(23)-C(14)-N(15)	121.283	H(24)-C(2)-C(1)	117.774
N(23)-C(14)-S(13)	122.598	C(3)-C(2)-C(1)	121.282
N(15)-C(14)-S(13)	116.031	C(7)-C(1)-C(6)	122.685
C(14)-S(13)-C(12)	88.807	C(7)-C(1)-C(2)	118.858
C(16)-C(12)-S(13)	108.914	C(6)-C(1)-C(2)	118.437
C(16)-C(12)-N(11)	138.014		

Table S3: Selected bond lengths (Å) of compound 3 using DFT-method from DMOL3 calculations.

Bond	Length(Å)	Bond	Length	Bond	Length(Å)
C(26)-H(42)	1.102	C(18)-C(19)	1.401	C(7)-O(9)	1.238
C(26)-H(41)	1.096	C(22)-C(17)	1.410	C(7)-C(8)	1.53
C(26)-H(40)	1.102	C(17)-C(18)	1.410	C(6)-H(30)	1.092
C(24)-C(26)	1.527	C(16)-C(17)	1.477	C(5)-H(29)	1.091
C(24)-O(25)	1.222	N(15)-C(16)	1.375	C(5)-C(6)	1.394
N(23)-H(39)	1.015	C(14)-N(23)	1.393	C(4)-N(10)	1.413
N(23)-C(24)	1.419	C(14)-N(15)	1.319	C(4)-C(5)	1.411
C(22)-H(38)	1.091	S(13)-C(14)	1.746	C(3)-H(28)	1.09
C(21)-H(37)	1.092	C(16)-C(12)	1.416	C(3)-C(4)	1.418
C(21)-C(22)	1.399	C(12)-S(13)	1.763	C(2)-H(27)	1.09
C(20)-H(36)	1.092	N(11)-C(12)	1.383	C(2)-C(3)	1.389
C(20)-C(21)	1.402	N(10)-N(11)	1.288	C(1)-C(7)	1.503
C(19)-H(35)	1.093	C(8)-H(33)	1.096	C(6)-C(1)	1.413
C(19)-C(20)	1.402	C(8)-H(32)	1.103	C(1)-C(2)	1.415
C(18)-H(34)	1.090	C(8)-H(31)	1.101		

Table S4: Selected bond angles (°) of compound 3 using DFT-method from DMOL3 calculations.

Angle	Degree(°)	Angle	Degree(°)
H(42)-C(26)-H(41)	109.412	N(23)-C(14)-S(13)	118.288
H(42)-C(26)-H(40)	107.829	N(15)-C(14)-S(13)	115.887
H(42)-C(26)-C(24)	112.121	C(14)-S(13)-C(12)	88.659
H(41)-C(26)-H(40)	108.861	C(16)-C(12)-S(13)	109.138
H(41)-C(26)-C(24)	108.293	C(16)-C(12)-N(11)	138.262
H(40)-C(26)-C(24)	110.277	S(13)-C(12)-N(11)	112.311
C(26)-C(24)-O(25)	124.345	C(12)-N(11)-N(10)	116.357
C(26)-C(24)-N(23)	111.932	N(11)-N(10)-C(4)	113.921
O(25)-C(24)-N(23)	123.718	H(33)-C(8)-H(32)	109.081
H(39)-N(23)-C(24)	115.706	H(33)-C(8)-H(31)	109.616
H(39)-N(23)-C(14)	114.924	H(33)-C(8)-C(7)	108.724
C(24)-N(23)-C(14)	129.294	H(32)-C(8)-H(31)	107.367
H(38)-C(22)-C(21)	119.881	H(32)-C(8)-C(7)	110.401
H(38)-C(22)-C(17)	119.878	H(31)-C(8)-C(7)	111.616
C(21)-C(22)-C(17)	120.238	O(9)-C(7)-C(8)	120.289
H(37)-C(21)-C(22)	119.612	O(9)-C(7)-C(1)	120.887
H(37)-C(21)-C(20)	120.200	C(8)-C(7)-C(1)	118.82
C(22)-C(21)-C(20)	120.186	H(30)-C(6)-C(5)	118.539
H(36)-C(20)-C(21)	119.886	H(30)-C(6)-C(1)	120.583
H(36)-C(20)-C(19)	120.175	C(5)-C(6)-C(1)	120.878
C(21)-C(20)-C(19)	119.939	H(29)-C(5)-C(6)	121.283
H(35)-C(19)-C(20)	120.261	H(29)-C(5)-C(4)	118.405
H(35)-C(19)-C(18)	119.659	C(6)-C(5)-C(4)	120.308
C(20)-C(19)-C(18)	120.08	N(10)-C(4)-C(5)	115.259
H(34)-C(18)-C(19)	120.558	N(10)-C(4)-C(3)	125.612
H(34)-C(18)-C(17)	119.216	C(5)-C(4)-C(3)	119.13
C(19)-C(18)-C(17)	120.194	H(28)-C(3)-C(4)	118.846
C(22)-C(17)-C(18)	119.293	H(28)-C(3)-C(2)	120.986
C(22)-C(17)-C(16)	121.021	C(4)-C(3)-C(2)	120.168
C(18)-C(17)-C(16)	119.685	H(27)-C(2)-C(3)	120.692
C(17)-C(16)-N(15)	118.135	H(27)-C(2)-C(1)	118.249
C(17)-C(16)-C(12)	126.976	C(3)-C(2)-C(1)	121.059
N(15)-C(16)-C(12)	114.883	C(7)-C(1)-C(6)	122.826
C(16)-N(15)-C(14)	111.377	C(7)-C(1)-C(2)	118.731
N(23)-C(14)-N(15)	125.825	C(6)-C(1)-C(2)	118.443

Table S5: Selected bond lengths (Å) of compound 4 using DFT-method from DMOL3 calculations.

Bond	Length(Å)	Bond	Length(Å)	Bond	Length(Å)
C(31)-H(49)	1.090	C(20)-H(41)	1.094	C(8)-H(38)	1.097
C(30)-H(48)	1.092	C(20)-C(21)	1.404	C(8)-H(37)	1.103
C(30)-C(31)	1.397	C(19)-H(40)	1.094	C(8)-H(36)	1.101
C(29)-H(47)	1.092	C(19)-C(20)	1.400	C(7)-O(9)	1.235
C(29)-C(30)	1.405	C(18)-H(39)	1.09	C(7)-C(8)	1.528
C(28)-H(46)	1.091	C(18)-C(19)	1.399	C(6)-H(35)	1.091
C(28)-C(29)	1.401	C(22)-C(17)	1.410	C(5)-H(34)	1.092
C(27)-H(45)	1.094	C(17)-C(18)	1.412	C(5)-C(6)	1.394
C(27)-C(28)	1.402	C(16)-C(17)	1.477	C(4)-N(10)	1.413
C(31)-C(26)	1.409	N(15)-C(16)	1.371	C(4)-C(5)	1.412
C(26)-C(27)	1.412	C(14)-N(23)	1.394	C(3)-H(33)	1.09
C(24)-C(26)	1.503	C(14)-N(15)	1.317	C(3)-C(4)	1.419
C(24)-O(25)	1.223	S(13)-C(14)	1.741	C(2)-H(32)	1.091
N(23)-H(44)	1.015	C(16)-C(12)	1.422	C(2)-C(3)	1.388
N(23)-C(24)	1.417	C(12)-S(13)	1.766	C(1)-C(7)	1.502
C(22)-H(43)	1.090	N(11)-C(12)	1.379	C(6)-C(1)	1.414
C(21)-H(42)	1.093	C(20)-H(41)	1.094	C(1)-C(2)	1.414
C(21)-C(22)	1.400	N(10)-N(11)	1.287		

Table S6: Selected bond angles (°) of compound 4 using DFT-method from DMOL3 calculations.

Angle	Degree(°)	Angle	Degree(°)
H(49)-C(31)-C(30)	121.392	C(18)-C(17)-C(16)	117.235
H(49)-C(31)-C(26)	118.456	C(17)-C(16)-N(15)	116.327
C(30)-C(31)-C(26)	120.150	C(17)-C(16)-C(12)	129.852
H(48)-C(30)-C(31)	119.362	N(15)-C(16)-C(12)	113.783
H(48)-C(30)-C(29)	120.086	C(16)-N(15)-C(14)	112.876
C(31)-C(30)-C(29)	120.386	N(23)-C(14)-N(15)	124.753
H(47)-C(29)-C(30)	119.984	N(23)-C(14)-S(13)	120.179
H(47)-C(29)-C(28)	120.158	N(15)-C(14)-S(13)	115.061
C(30)-C(29)-C(28)	119.818	C(14)-S(13)-C(12)	88.984
H(46)-C(28)-C(29)	120.265	C(16)-C(12)-S(13)	109.205
H(46)-C(28)-C(27)	119.825	C(16)-C(12)-N(11)	138.717
C(29)-C(28)-C(27)	119.908	S(13)-C(12)-N(11)	112.02
H(45)-C(27)-C(28)	118.795	C(12)-N(11)-N(10)	117.026
H(45)-C(27)-C(26)	120.704	N(11)-N(10)-C(4)	114.144
C(28)-C(27)-C(26)	120.415	H(38)-C(8)-H(37)	109.249
C(31)-C(26)-C(27)	119.170	H(38)-C(8)-H(36)	109.49
C(31)-C(26)-C(24)	116.659	H(38)-C(8)-C(7)	109.011
C(27)-C(26)-C(24)	123.871	H(37)-C(8)-H(36)	107.208
C(26)-C(24)-O(25)	121.948	H(37)-C(8)-C(7)	110.469
C(26)-C(24)-N(23)	114.466	H(36)-C(8)-C(7)	111.379
O(25)-C(24)-N(23)	123.569	O(9)-C(7)-C(8)	120.513
H(44)-N(23)-C(24)	116.262	O(9)-C(7)-C(1)	120.408
H(44)-N(23)-C(14)	116.001	C(8)-C(7)-C(1)	118.934
C(24)-N(23)-C(14)	126.898	H(35)-C(6)-C(5)	118.844
H(43)-C(22)-C(21)	119.950	H(35)-C(6)-C(1)	120.508
H(43)-C(22)-C(17)	120.345	C(5)-C(6)-C(1)	120.647
C(21)-C(22)-C(17)	119.696	H(34)-C(5)-C(6)	121.04
H(42)-C(21)-C(22)	118.995	H(34)-C(5)-C(4)	118.206
H(42)-C(21)-C(20)	119.628	C(6)-C(5)-C(4)	120.605
C(22)-C(21)-C(20)	121.376	N(10)-C(4)-C(5)	115.531
H(41)-C(20)-C(21)	120.39	N(10)-C(4)-C(3)	125.582
H(41)-C(20)-C(19)	120.261	C(5)-C(4)-C(3)	118.886
C(21)-C(20)-C(19)	119.346	H(33)-C(3)-C(4)	118.759
H(40)-C(19)-C(20)	120.544	H(33)-C(3)-C(2)	121.191
H(40)-C(19)-C(18)	120.001	C(4)-C(3)-C(2)	120.047

C(20)-C(19)-C(18)	119.441	H(32)-C(2)-C(3)	120.864
H(39)-C(18)-C(19)	119.848	H(32)-C(2)-C(1)	117.76
H(39)-C(18)-C(17)	118.424	C(3)-C(2)-C(1)	121.374
C(19)-C(18)-C(17)	121.716	C(7)-C(1)-C(6)	123.715
C(22)-C(17)-C(18)	118.381	C(7)-C(1)-C(2)	117.96
C(22)-C(17)-C(16)	124.082	C(6)-C(1)-C(2)	118.315
H(49)-C(31)-C(30)	121.392	C(18)-C(17)-C(16)	117.235
H(49)-C(31)-C(26)	118.456	C(17)-C(16)-N(15)	116.327
C(30)-C(31)-C(26)	120.15	C(17)-C(16)-C(12)	129.852
H(48)-C(30)-C(31)	119.362	N(15)-C(16)-C(12)	113.783
H(48)-C(30)-C(29)	120.086	C(16)-N(15)-C(14)	112.876
C(31)-C(30)-C(29)	120.386	N(23)-C(14)-N(15)	124.753
H(47)-C(29)-C(30)	119.984	N(23)-C(14)-S(13)	120.179
H(47)-C(29)-C(28)	120.158	N(15)-C(14)-S(13)	115.061
C(30)-C(29)-C(28)	119.818	C(14)-S(13)-C(12)	88.984
H(46)-C(28)-C(29)	120.265	C(16)-C(12)-S(13)	109.205
H(46)-C(28)-C(27)	119.825	C(16)-C(12)-N(11)	138.717
C(29)-C(28)-C(27)	119.908	S(13)-C(12)-N(11)	112.02
H(45)-C(27)-C(28)	118.795	C(12)-N(11)-N(10)	117.026
H(45)-C(27)-C(26)	120.704	N(11)-N(10)-C(4)	114.144
C(28)-C(27)-C(26)	120.415	H(38)-C(8)-H(37)	109.249
C(31)-C(26)-C(27)	119.17	H(38)-C(8)-H(36)	109.49
C(31)-C(26)-C(24)	116.659	H(38)-C(8)-C(7)	109.011
C(27)-C(26)-C(24)	123.871	H(37)-C(8)-H(36)	107.208
C(26)-C(24)-O(25)	121.948	H(37)-C(8)-C(7)	110.469
C(26)-C(24)-N(23)	114.466	H(36)-C(8)-C(7)	111.379
O(25)-C(24)-N(23)	123.569	O(9)-C(7)-C(8)	120.513
H(44)-N(23)-C(24)	116.262	O(9)-C(7)-C(1)	120.408
H(44)-N(23)-C(14)	116.001	C(8)-C(7)-C(1)	118.934
C(24)-N(23)-C(14)	126.898	H(35)-C(6)-C(5)	118.844
H(43)-C(22)-C(21)	119.95	H(35)-C(6)-C(1)	120.508
H(43)-C(22)-C(17)	120.345	C(5)-C(6)-C(1)	120.647
C(21)-C(22)-C(17)	119.696	H(34)-C(5)-C(6)	121.04
H(42)-C(21)-C(22)	118.995	H(34)-C(5)-C(4)	118.206
H(42)-C(21)-C(20)	119.628	C(6)-C(5)-C(4)	120.605
C(22)-C(21)-C(20)	121.376	N(10)-C(4)-C(5)	115.531
H(41)-C(20)-C(21)	120.39	N(10)-C(4)-C(3)	125.582
H(41)-C(20)-C(19)	120.261	C(5)-C(4)-C(3)	118.886
C(21)-C(20)-C(19)	119.346	H(33)-C(3)-C(4)	118.759
H(40)-C(19)-C(20)	120.544	H(33)-C(3)-C(2)	121.191
H(40)-C(19)-C(18)	120.001	C(4)-C(3)-C(2)	120.047
C(20)-C(19)-C(18)	119.441	H(32)-C(2)-C(3)	120.864
H(39)-C(18)-C(19)	119.848	H(32)-C(2)-C(1)	117.76
H(39)-C(18)-C(17)	118.424	C(3)-C(2)-C(1)	121.374
C(19)-C(18)-C(17)	121.716	C(7)-C(1)-C(6)	123.715
C(22)-C(17)-C(18)	118.381	C(7)-C(1)-C(2)	117.96

Table S7: Selected bond lengths (Å) of compound 5 using DFT-method from DMOL3 calculations.

Bond	Length(Å)	Bond	Length(Å)	Bond	Length(Å)
C(26)-H(42)	1.100	C(18)-C(19)	1.398	C(7)-O(9)	1.239
C(26)-H(41)	1.098	C(22)-C(17)	1.408	C(7)-C(8)	1.530
C(26)-Cl(27)	1.791	C(17)-C(18)	1.408	C(6)-H(31)	1.091
C(24)-C(26)	1.537	C(16)-C(17)	1.479	C(5)-H(30)	1.091
C(24)-O(25)	1.229	N(15)-C(16)	1.373	C(5)-C(6)	1.394
N(23)-H(40)	1.016	C(14)-N(23)	1.393	C(4)-N(10)	1.414
N(23)-C(24)	1.389	C(14)-N(15)	1.328	C(4)-C(5)	1.41
C(22)-H(39)	1.090	S(13)-C(14)	1.727	C(3)-H(29)	1.089
C(21)-H(38)	1.092	C(16)-C(12)	1.413	C(3)-C(4)	1.416
C(21)-C(22)	1.395	C(12)-S(13)	1.765	C(2)-H(28)	1.090
C(20)-H(37)	1.092	N(11)-C(12)	1.382	C(2)-C(3)	1.388
C(20)-C(21)	1.403	N(10)-N(11)	1.289	C(1)-C(7)	1.502
C(19)-H(36)	1.092	C(8)-H(34)	1.095	C(6)-C(1)	1.411
C(19)-C(20)	1.402	C(8)-H(33)	1.102	C(1)-C(2)	1.414
C(18)-H(35)	1.091	C(8)-H(32)	1.100		

Table S8: Selected bond angles (°) of compound 5 using DFT-method from DMOL3 calculations.

Angle	Degree(°)	Angle	Degree(°)
H(42)-C(26)-H(41)	108.745	N(23)-C(14)-S(13)	123.648
H(42)-C(26)-Cl(27)	107.837	N(15)-C(14)-S(13)	117.219
H(42)-C(26)-C(24)	108.512	C(14)-S(13)-C(12)	87.621
H(41)-C(26)-Cl(27)	107.853	C(16)-C(12)-S(13)	110.410
H(41)-C(26)-C(24)	111.360	C(16)-C(12)-N(11)	136.574
Cl(27)-C(26)-C(24)	112.418	S(13)-C(12)-N(11)	112.892
C(26)-C(24)-O(25)	124.859	C(12)-N(11)-N(10)	116.189
C(26)-C(24)-N(23)	113.056	N(11)-N(10)-C(4)	114.912
O(25)-C(24)-N(23)	122.036	H(34)-C(8)-H(33)	109.136
H(40)-N(23)-C(24)	120.996	H(34)-C(8)-H(32)	109.246
H(40)-N(23)-C(14)	113.403	H(34)-C(8)-C(7)	108.638
C(24)-N(23)-C(14)	125.567	H(33)-C(8)-H(32)	107.301
H(39)-C(22)-C(21)	119.856	H(33)-C(8)-C(7)	110.626
H(39)-C(22)-C(17)	119.824	H(32)-C(8)-C(7)	111.853
C(21)-C(22)-C(17)	120.289	O(9)-C(7)-C(8)	120.399
H(38)-C(21)-C(22)	119.829	O(9)-C(7)-C(1)	120.498
H(38)-C(21)-C(20)	120.358	C(8)-C(7)-C(1)	119.101
C(22)-C(21)-C(20)	119.812	H(31)-C(6)-C(5)	118.866
H(37)-C(20)-C(21)	119.809	H(31)-C(6)-C(1)	120.496
H(37)-C(20)-C(19)	120.018	C(5)-C(6)-C(1)	120.623
C(21)-C(20)-C(19)	120.165	H(30)-C(5)-C(6)	121.614
H(36)-C(19)-C(20)	120.374	H(30)-C(5)-C(4)	117.721
H(36)-C(19)-C(18)	119.449	C(6)-C(5)-C(4)	120.645
C(20)-C(19)-C(18)	120.162	N(10)-C(4)-C(5)	115.267
H(35)-C(18)-C(19)	120.612	N(10)-C(4)-C(3)	125.747
H(35)-C(18)-C(17)	119.540	C(5)-C(4)-C(3)	118.973
C(19)-C(18)-C(17)	119.839	H(29)-C(3)-C(4)	118.916
C(22)-C(17)-C(18)	119.713	H(29)-C(3)-C(2)	121.041
C(22)-C(17)-C(16)	120.836	C(4)-C(3)-C(2)	120.042
C(18)-C(17)-C(16)	119.444	H(28)-C(2)-C(3)	120.994
C(17)-C(16)-N(15)	117.954	H(28)-C(2)-C(1)	117.699
C(17)-C(16)-C(12)	128.092	C(3)-C(2)-C(1)	121.289
N(15)-C(16)-C(12)	113.92	C(7)-C(1)-C(6)	123.055
C(16)-N(15)-C(14)	110.786	C(7)-C(1)-C(2)	118.532
N(23)-C(14)-N(15)	119.113	C(6)-C(1)-C(2)	118.41
H(42)-C(26)-H(41)	108.745	N(23)-C(14)-S(13)	123.648
H(42)-C(26)-Cl(27)	107.837	N(15)-C(14)-S(13)	117.219
H(42)-C(26)-C(24)	108.512	C(14)-S(13)-C(12)	87.621

H(41)-C(26)-Cl(27)	107.853	C(16)-C(12)-S(13)	110.41
H(41)-C(26)-C(24)	111.36	C(16)-C(12)-N(11)	136.574
Cl(27)-C(26)-C(24)	112.418	S(13)-C(12)-N(11)	112.892
C(26)-C(24)-O(25)	124.859	C(12)-N(11)-N(10)	116.189
C(26)-C(24)-N(23)	113.056	N(11)-N(10)-C(4)	114.912
O(25)-C(24)-N(23)	122.036	H(34)-C(8)-H(33)	109.136
H(40)-N(23)-C(24)	120.996	H(34)-C(8)-H(32)	109.246
H(40)-N(23)-C(14)	113.403	H(34)-C(8)-C(7)	108.638
C(24)-N(23)-C(14)	125.567	H(33)-C(8)-H(32)	107.301
H(39)-C(22)-C(21)	119.856	H(33)-C(8)-C(7)	110.626
H(39)-C(22)-C(17)	119.824	H(32)-C(8)-C(7)	111.853
C(21)-C(22)-C(17)	120.289	O(9)-C(7)-C(8)	120.399
H(38)-C(21)-C(22)	119.829	O(9)-C(7)-C(1)	120.498
H(38)-C(21)-C(20)	120.358	C(8)-C(7)-C(1)	119.101
C(22)-C(21)-C(20)	119.812	H(31)-C(6)-C(5)	118.866
H(37)-C(20)-C(21)	119.809	H(31)-C(6)-C(1)	120.496
H(37)-C(20)-C(19)	120.018	C(5)-C(6)-C(1)	120.623
C(21)-C(20)-C(19)	120.165	H(30)-C(5)-C(6)	121.614
H(36)-C(19)-C(20)	120.374	H(30)-C(5)-C(4)	117.721
H(36)-C(19)-C(18)	119.449	C(6)-C(5)-C(4)	120.645
C(20)-C(19)-C(18)	120.162	N(10)-C(4)-C(5)	115.267
H(35)-C(18)-C(19)	120.612	N(10)-C(4)-C(3)	125.747
H(35)-C(18)-C(17)	119.540	C(5)-C(4)-C(3)	118.973
C(19)-C(18)-C(17)	119.839	H(29)-C(3)-C(4)	118.916
C(22)-C(17)-C(18)	119.713	H(29)-C(3)-C(2)	121.041
C(22)-C(17)-C(16)	120.836	C(4)-C(3)-C(2)	120.042
C(18)-C(17)-C(16)	119.444	H(28)-C(2)-C(3)	120.994
C(17)-C(16)-N(15)	117.954	H(28)-C(2)-C(1)	117.699
C(17)-C(16)-C(12)	128.092	C(3)-C(2)-C(1)	121.289
N(15)-C(16)-C(12)	113.92	C(7)-C(1)-C(6)	123.055
C(16)-N(15)-C(14)	110.786	C(7)-C(1)-C(2)	118.532

Table S9: Selected bond lengths (Å) of compound 6 using DFT-method from DMOL3 calculations.

Bond	Length(Å)	Bond	Length(Å)	Bond	Length(Å)
C(36)-H(55)	1.092	N(23)-H(49)	1.018	N(11)-C(12)	1.389
C(35)-H(54)	1.092	N(23)-C(24)	1.386	N(10)-N(11)	1.287
C(35)-C(36)	1.397	C(22)-H(48)	1.088	C(8)-H(43)	1.098
C(34)-H(53)	1.093	C(21)-H(47)	1.093	C(8)-H(42)	1.102
C(34)-C(35)	1.410	C(21)-C(22)	1.396	C(8)-H(41)	1.102
C(33)-H(52)	1.091	C(20)-H(46)	1.092	C(7)-O(9)	1.238
C(33)-C(34)	1.401	C(20)-C(21)	1.405	C(7)-C(8)	1.526
C(36)-C(31)	1.408	C(19)-H(45)	1.092	C(6)-H(40)	1.092
C(31)-N(32)	1.393	C(19)-C(20)	1.402	C(5)-H(39)	1.091
C(30)-C(33)	1.405	C(18)-H(44)	1.091	C(5)-C(6)	1.393
C(30)-C(31)	1.423	C(18)-C(19)	1.398	C(4)-N(10)	1.412
S(29)-C(30)	1.751	C(22)-C(17)	1.411	C(4)-C(5)	1.413
C(28)-N(32)	1.308	C(17)-C(18)	1.412	C(3)-H(38)	1.090
C(28)-S(29)	1.783	C(16)-C(17)	1.478	C(3)-C(4)	1.417
S(27)-C(28)	1.761	N(15)-C(16)	1.376	C(2)-H(37)	1.091
C(26)-H(51)	1.095	C(14)-N(23)	1.394	C(2)-C(3)	1.389
C(26)-H(50)	1.101	C(14)-N(15)	1.323	C(1)-C(7)	1.503
C(26)-S(27)	1.849	S(13)-C(14)	1.730	C(6)-C(1)	1.412
C(24)-C(26)	1.527	C(16)-C(12)	1.416		

Table S10: Selected bond angles (°) of compound 6 using DFT-method from DMOL3 calculations.

Angle	Degree(°)	Angle	Degree(°)
H(55)-C(36)-C(35)	121.753	C(20)-C(19)-C(18)	120.063
H(55)-C(36)-C(31)	119.302	H(44)-C(18)-C(19)	120.472
C(35)-C(36)-C(31)	118.945	H(44)-C(18)-C(17)	118.819
H(54)-C(35)-C(36)	119.535	C(19)-C(18)-C(17)	120.707
H(54)-C(35)-C(34)	119.569	C(22)-C(17)-C(18)	118.711
C(36)-C(35)-C(34)	120.896	C(22)-C(17)-C(16)	122.539
H(53)-C(34)-C(35)	119.508	C(18)-C(17)-C(16)	118.705
H(53)-C(34)-C(33)	119.288	C(17)-C(16)-N(15)	117.291
C(35)-C(34)-C(33)	121.204	C(17)-C(16)-C(12)	129.046
H(52)-C(33)-C(34)	120.919	N(15)-C(16)-C(12)	113.656
H(52)-C(33)-C(30)	121.223	C(16)-N(15)-C(14)	111.322
C(34)-C(33)-C(30)	117.858	N(23)-C(14)-N(15)	119.308
C(31)-N(32)-C(28)	110.258	N(23)-C(14)-S(13)	123.741
C(36)-C(31)-N(32)	124.69	N(15)-C(14)-S(13)	116.889
C(36)-C(31)-C(30)	119.654	C(14)-S(13)-C(12)	87.822
N(32)-C(31)-C(30)	115.650	C(16)-C(12)-S(13)	110.155
C(33)-C(30)-C(31)	121.442	C(16)-C(12)-N(11)	136.474
C(33)-C(30)-S(29)	129.150	S(13)-C(12)-N(11)	112.879
C(31)-C(30)-S(29)	109.408	C(12)-N(11)-N(10)	114.365
C(30)-S(29)-C(28)	88.313	N(11)-N(10)-C(4)	115.447
N(32)-C(28)-S(29)	116.368	H(43)-C(8)-H(42)	109.288
N(32)-C(28)-S(27)	125.409	H(43)-C(8)-H(41)	109.226
S(29)-C(28)-S(27)	118.215	H(43)-C(8)-C(7)	108.775
C(28)-S(27)-C(26)	98.959	H(42)-C(8)-H(41)	107.040
H(51)-C(26)-H(50)	108.872	H(42)-C(8)-C(7)	111.743
H(51)-C(26)-S(27)	110.211	H(41)-C(8)-C(7)	110.728
H(51)-C(26)-C(24)	108.361	O(9)-C(7)-C(8)	120.174
H(50)-C(26)-S(27)	107.603	O(9)-C(7)-C(1)	120.929
H(50)-C(26)-C(24)	111.663	C(8)-C(7)-C(1)	118.897
S(27)-C(26)-C(24)	110.125	H(40)-C(6)-C(5)	119.098
C(26)-C(24)-O(25)	122.865	H(40)-C(6)-C(1)	120.681
C(26)-C(24)-N(23)	114.920	C(5)-C(6)-C(1)	120.220
O(25)-C(24)-N(23)	122.211	H(39)-C(5)-C(6)	121.313
H(49)-N(23)-C(24)	119.484	H(39)-C(5)-C(4)	118.004
H(49)-N(23)-C(14)	114.435	C(6)-C(5)-C(4)	120.683
C(24)-N(23)-C(14)	125.626	N(10)-C(4)-C(5)	114.069
H(48)-C(22)-C(21)	119.591	N(10)-C(4)-C(3)	126.481
H(48)-C(22)-C(17)	119.883	C(5)-C(4)-C(3)	119.446
C(21)-C(22)-C(17)	120.521	H(38)-C(3)-C(4)	118.924
H(47)-C(21)-C(22)	119.642	H(38)-C(3)-C(2)	121.597
H(47)-C(21)-C(20)	120.060	C(4)-C(3)-C(2)	119.472
C(22)-C(21)-C(20)	120.297	H(37)-C(2)-C(3)	120.796
H(46)-C(20)-C(21)	120.282	H(37)-C(2)-C(1)	117.781
H(46)-C(20)-C(19)	120.031	C(3)-C(2)-C(1)	121.422
C(21)-C(20)-C(19)	119.687	C(7)-C(1)-C(6)	122.606
H(45)-C(19)-C(20)	120.325	C(7)-C(1)-C(2)	118.622
H(45)-C(19)-C(18)	119.611	C(6)-C(1)-C(2)	118.755

Table S11: Selected bond lengths (Å) of compound 8a using DFT-method from DMOL3 calculations.

Bond	Length(Å)	Bond	Length(Å)	Bond	Length(Å)
C(25)-N(26)	1.159	N(16)-N(17)	1.234	C(7)-H(28)	1.103
C(23)-H(38)	1.104	C(15)-C(25)	1.322	C(7)-C(8)	1.343
C(22)-H(37)	1.103	C(15)-N(16)	1.270	C(11)-C(6)	1.349
C(22)-C(23)	1.343	C(13)-C(15)	1.368	C(6)-C(7)	1.350
C(21)-Cl(24)	1.727	C(13)-O(14)	1.211	C(4)-N(12)	1.349
C(21)-C(22)	1.342	N(12)-H(33)	1.010	N(5)-C(4)	1.265
C(20)-H(36)	1.103	N(12)-C(13)	1.370	S(3)-C(4)	1.460
C(20)-C(21)	1.342	C(11)-H(32)	1.101	C(2)-H(27)	1.097
C(19)-H(35)	1.103	C(10)-H(31)	1.103	C(2)-S(3)	1.464
C(19)-C(20)	1.343	C(10)-C(11)	1.343	C(1)-C(6)	1.350
C(23)-C(18)	1.345	C(9)-H(30)	1.103	N(5)-C(1)	1.266
C(18)-C(19)	1.346	C(9)-C(10)	1.340	C(1)-C(2)	1.345
N(17)-H(34)	1.049	C(8)-H(29)	1.103	N(17)-C(18)	1.272
C(8)-C(9)	1.340				

Table S12: Selected bond angles (°) of compound 8a using DFT-method from DMOL3 calculations.

Angle	Degree(°)	Angle	Degree(°)
N(26)-C(25)-C(15)	178.330	C(13)-N(12)-C(4)	133.188
H(38)-C(23)-C(22)	118.540	H(32)-C(11)-C(10)	116.318
H(38)-C(23)-C(18)	120.623	H(32)-C(11)-C(6)	121.901
C(22)-C(23)-C(18)	120.837	C(10)-C(11)-C(6)	121.781
H(37)-C(22)-C(23)	119.331	H(31)-C(10)-C(11)	120.127
H(37)-C(22)-C(21)	120.712	H(31)-C(10)-C(9)	119.617
C(23)-C(22)-C(21)	119.956	C(11)-C(10)-C(9)	120.256
Cl(24)-C(21)-C(22)	120.104	H(30)-C(9)-C(10)	120.462
Cl(24)-C(21)-C(20)	120.161	H(30)-C(9)-C(8)	120.454
C(22)-C(21)-C(20)	119.735	C(10)-C(9)-C(8)	119.085
H(36)-C(20)-C(21)	120.644	H(29)-C(8)-C(9)	119.697
H(36)-C(20)-C(19)	119.317	H(29)-C(8)-C(7)	120.141
C(21)-C(20)-C(19)	120.039	C(9)-C(8)-C(7)	120.163
H(35)-C(19)-C(20)	118.127	H(28)-C(7)-C(8)	116.603
H(35)-C(19)-C(18)	121.144	H(28)-C(7)-C(6)	121.534
C(20)-C(19)-C(18)	120.73	C(8)-C(7)-C(6)	121.863
C(23)-C(18)-C(19)	118.703	C(11)-C(6)-C(7)	116.852
C(23)-C(18)-N(17)	117.864	C(11)-C(6)-C(1)	122.546
C(19)-C(18)-N(17)	123.433	C(7)-C(6)-C(1)	120.602
H(34)-N(17)-C(18)	115.380	C(4)-N(5)-C(1)	111.309
H(34)-N(17)-N(16)	116.198	N(12)-C(4)-N(5)	117.544
C(18)-N(17)-N(16)	128.423	N(12)-C(4)-S(3)	128.134
N(17)-N(16)-C(15)	121.664	N(5)-C(4)-S(3)	114.321
C(25)-C(15)-N(16)	120.593	C(4)-S(3)-C(2)	94.265
C(25)-C(15)-C(13)	119.748	H(27)-C(2)-S(3)	120.752
N(16)-C(15)-C(13)	119.659	H(27)-C(2)-C(1)	127.138
C(15)-C(13)-O(14)	123.396	S(3)-C(2)-C(1)	112.109
C(15)-C(13)-N(12)	113.877	C(6)-C(1)-N(5)	124.037
O(14)-C(13)-N(12)	122.726	C(6)-C(1)-C(2)	127.967
H(33)-N(12)-C(13)	118.277	N(5)-C(1)-C(2)	107.996
H(33)-N(12)-C(4)	108.535		

Table S13: Selected bond lengths (Å) of compound 8b using DFT-method from DMOL3 calculations.

Bond	Length(Å)	Bond	Length(Å)	Bond	Length(Å)
N(26)-O(28)	1.245	N(17)-H(36)	1.027	C(8)-H(31)	1.092
N(26)-O(27)	1.246	N(17)-C(18)	1.405	C(8)-C(9)	1.402
C(24)-N(25)	1.176	N(16)-N(17)	1.319	C(7)-H(30)	1.09
C(23)-H(40)	1.092	C(15)-C(24)	1.434	C(7)-C(8)	1.400
C(22)-H(39)	1.089	C(15)-N(16)	1.322	C(11)-C(6)	1.414
C(22)-C(23)	1.394	C(13)-C(15)	1.496	C(6)-C(7)	1.411
C(21)-N(26)	1.480	C(13)-O(14)	1.236	C(4)-N(12)	1.397
C(21)-C(22)	1.403	N(12)-H(35)	1.017	N(5)-C(4)	1.315
C(20)-H(38)	1.088	N(12)-C(13)	1.386	S(3)-C(4)	1.747
C(20)-C(21)	1.405	C(11)-H(34)	1.090	C(2)-H(29)	1.088
C(19)-H(37)	1.090	C(10)-H(33)	1.091	C(2)-S(3)	1.726
C(19)-C(20)	1.393	C(10)-C(11)	1.398	C(1)-C(6)	1.478
C(23)-C(18)	1.413	C(9)-H(32)	1.091	N(5)-C(1)	1.387
C(18)-C(19)	1.410	C(9)-C(10)	1.402	C(1)-C(2)	1.389

Table S14: Selected bond angles (°) of compound 8a using DFT-method from DMOL3 calculations.

Angle	Degree(°)	Angle	Degree(°)
O(28)-N(26)-O(27)	124.599	H(35)-N(12)-C(13)	120.505
O(28)-N(26)-C(21)	117.702	H(35)-N(12)-C(4)	114.542
O(27)-N(26)-C(21)	117.698	C(13)-N(12)-C(4)	124.953
N(25)-C(24)-C(15)	178.327	H(34)-C(11)-C(10)	118.913
H(40)-C(23)-C(22)	120.172	H(34)-C(11)-C(6)	120.281
H(40)-C(23)-C(18)	120.320	C(10)-C(11)-C(6)	120.795
C(22)-C(23)-C(18)	119.49	H(33)-C(10)-C(11)	119.246
H(39)-C(22)-C(23)	120.992	H(33)-C(10)-C(9)	119.922
H(39)-C(22)-C(21)	119.573	C(11)-C(10)-C(9)	120.83
C(23)-C(22)-C(21)	119.420	H(32)-C(9)-C(10)	120.28
N(26)-C(21)-C(22)	119.466	H(32)-C(9)-C(8)	120.739
N(26)-C(21)-C(20)	119.141	C(10)-C(9)-C(8)	118.981
C(22)-C(21)-C(20)	121.387	H(31)-C(8)-C(9)	120.079
H(38)-C(20)-C(21)	119.635	H(31)-C(8)-C(7)	119.608
H(38)-C(20)-C(19)	120.945	C(9)-C(8)-C(7)	120.267
C(21)-C(20)-C(19)	119.391	H(30)-C(7)-C(8)	119.889
H(37)-C(19)-C(20)	120.687	H(30)-C(7)-C(6)	118.779
H(37)-C(19)-C(18)	119.732	C(8)-C(7)-C(6)	121.289
C(20)-C(19)-C(18)	119.578	C(11)-C(6)-C(7)	117.79
C(23)-C(18)-C(19)	120.722	C(11)-C(6)-C(1)	122.386
C(23)-C(18)-N(17)	118.635	C(7)-C(6)-C(1)	119.824
C(19)-C(18)-N(17)	120.641	C(4)-N(5)-C(1)	110.465
H(36)-N(17)-C(18)	118.368	N(12)-C(4)-N(5)	120.776
H(36)-N(17)-N(16)	120.837	N(12)-C(4)-S(3)	123.136
C(18)-N(17)-N(16)	120.788	N(5)-C(4)-S(3)	116.011
N(17)-N(16)-C(15)	120.91	C(4)-S(3)-C(2)	87.954
C(24)-C(15)-N(16)	121.258	H(29)-C(2)-S(3)	119.102
C(24)-C(15)-C(13)	122.435	H(29)-C(2)-C(1)	129.292
N(16)-C(15)-C(13)	116.287	S(3)-C(2)-C(1)	111.448
C(15)-C(13)-O(14)	123.071	C(6)-C(1)-N(5)	118.95
C(15)-C(13)-N(12)	114.528	C(6)-C(1)-C(2)	127.142
O(14)-C(13)-N(12)	122.267	N(5)-C(1)-C(2)	113.856

Table S15: Selected bond lengths (Å) of compound 8c using DFT-method from DMOL3 calculations.

Bond	Length(Å)	Bond	Length(Å)	Bond	Length(Å)
N(26)-O(28)	1.480	N(17)-H(36)	1.110	C(8)-H(31)	1.14
N(26)-O(27)	1.480	N(17)-C(18)	1.510	C(8)-C(9)	1.539
C(24)-N(25)	1.509	N(16)-N(17)	1.481	C(7)-H(30)	1.14
C(23)-H(40)	1.140	C(15)-C(24)	1.539	C(7)-C(8)	1.54
C(22)-H(39)	1.140	C(15)-N(16)	1.509	C(11)-C(6)	1.54
C(22)-C(23)	1.539	C(13)-C(15)	1.540	C(6)-C(7)	1.541
C(21)-N(26)	1.509	C(13)-O(14)	1.510	C(4)-N(12)	1.513
C(21)-C(22)	1.539	N(12)-H(35)	1.110	N(5)-C(4)	1.523
C(20)-H(38)	1.140	N(12)-C(13)	1.513	S(3)-C(4)	1.796
C(20)-C(21)	1.541	C(11)-H(34)	1.140	C(2)-H(29)	1.140
C(19)-H(37)	1.140	C(10)-H(33)	1.140	C(2)-S(3)	1.790
C(19)-C(20)	1.542	C(10)-C(11)	1.540	C(1)-C(6)	1.540
C(23)-C(18)	1.539	C(9)-H(32)	1.140	N(5)-C(1)	1.532
C(18)-C(19)	1.540	C(9)-C(10)	1.541	C(1)-C(2)	1.545

Table S16: Selected bond angles (°) of compound 8c using DFT-method from DMOL3 calculations.

Angle	Degree(°)	Angle	Degree(°)
O(28)-N(26)-O(27)	120.02	H(35)-N(12)-C(13)	118.796
O(28)-N(26)-C(21)	119.894	H(35)-N(12)-C(4)	118.863
O(27)-N(26)-C(21)	120.087	C(13)-N(12)-C(4)	122.341
N(25)-C(24)-C(15)	179.915	H(34)-C(11)-C(10)	120.093
H(40)-C(23)-C(22)	119.941	H(34)-C(11)-C(6)	120.059
H(40)-C(23)-C(18)	119.919	C(10)-C(11)-C(6)	119.848
C(22)-C(23)-C(18)	120.139	H(33)-C(10)-C(11)	119.957
H(39)-C(22)-C(23)	120.007	H(33)-C(10)-C(9)	119.934
H(39)-C(22)-C(21)	120.036	C(11)-C(10)-C(9)	120.109
C(23)-C(22)-C(21)	119.957	H(32)-C(9)-C(10)	120.002
N(26)-C(21)-C(22)	119.76	H(32)-C(9)-C(8)	119.982
N(26)-C(21)-C(20)	120.251	C(10)-C(9)-C(8)	120.016
C(22)-C(21)-C(20)	119.989	H(31)-C(8)-C(9)	120.041
H(38)-C(20)-C(21)	119.968	H(31)-C(8)-C(7)	120.071
H(38)-C(20)-C(19)	119.985	C(9)-C(8)-C(7)	119.888
C(21)-C(20)-C(19)	120.047	H(30)-C(7)-C(8)	119.976
H(37)-C(19)-C(20)	120.072	H(30)-C(7)-C(6)	119.905
H(37)-C(19)-C(18)	120.066	C(8)-C(7)-C(6)	120.119
C(20)-C(19)-C(18)	119.862	C(11)-C(6)-C(7)	120.019
C(23)-C(18)-C(19)	120.005	C(11)-C(6)-C(1)	120.274
C(23)-C(18)-N(17)	119.346	C(7)-C(6)-C(1)	119.707
C(19)-C(18)-N(17)	120.649	C(4)-N(5)-C(1)	111.584
H(36)-N(17)-C(18)	119.422	N(12)-C(4)-N(5)	123.803
H(36)-N(17)-N(16)	119.412	N(12)-C(4)-S(3)	126.601
C(18)-N(17)-N(16)	121.166	N(5)-C(4)-S(3)	109.596
N(17)-N(16)-C(15)	118.064	C(4)-S(3)-C(2)	97.663
C(24)-C(15)-N(16)	119.272	H(29)-C(2)-S(3)	126.018
C(24)-C(15)-C(13)	119.277	H(29)-C(2)-C(1)	125.954
N(16)-C(15)-C(13)	121.451	S(3)-C(2)-C(1)	108.029
C(15)-C(13)-O(14)	120.384	C(6)-C(1)-N(5)	123.025
C(15)-C(13)-N(12)	118.262	C(6)-C(1)-C(2)	123.846
O(14)-C(13)-N(12)	121.354	N(5)-C(1)-C(2)	113.129

Table S17: Selected bond lengths (Å) of compound 8d using DFT-method from DMOL3 calculations.

Bond	Length(Å)	Bond	Length(Å)	Bond	Length(Å)
C(25)-N(26)	1.178	N(17)-C(18)	1.420	C(8)-C(9)	1.402
O(24)-H(39)	0.974	N(16)-N(17)	1.309	C(7)-H(28)	1.089
C(23)-H(38)	1.093	C(15)-C(25)	1.428	C(7)-C(8)	1.402
C(22)-H(37)	1.093	C(15)-N(16)	1.325	C(11)-C(6)	1.412
C(22)-C(23)	1.400	C(13)-C(15)	1.491	C(6)-C(7)	1.413
C(21)-O(24)	1.384	C(13)-O(14)	1.240	C(4)-N(12)	1.397
C(21)-C(22)	1.404	N(12)-H(33)	1.017	N(5)-C(4)	1.314
C(20)-H(36)	1.091	N(12)-C(13)	1.388	S(3)-C(4)	1.750
C(20)-C(21)	1.406	C(11)-H(32)	1.091	C(2)-H(27)	1.087
C(19)-H(35)	1.089	C(10)-H(31)	1.091	C(2)-S(3)	1.724
C(19)-C(20)	1.393	C(10)-C(11)	1.399	C(1)-C(6)	1.476
C(23)-C(18)	1.403	C(9)-H(30)	1.091	N(5)-C(1)	1.39
C(18)-C(19)	1.408	C(9)-C(10)	1.404	C(1)-C(2)	1.387
N(17)-H(34)	1.026	C(8)-H(29)	1.092		

Table S18: Selected bond angles (°) of compound 8d using DFT-method from DMOL3 calculations.

Angle	Degree(°)	Angle	Degree(°)
N(26)-C(25)-C(15)	175.262	H(33)-N(12)-C(4)	115.661
H(39)-O(24)-C(21)	108.621	C(13)-N(12)-C(4)	123.382
H(38)-C(23)-C(22)	119.641	H(32)-C(11)-C(10)	118.769
H(38)-C(23)-C(18)	120.506	H(32)-C(11)-C(6)	119.971
C(22)-C(23)-C(18)	119.852	C(10)-C(11)-C(6)	121.260
H(37)-C(22)-C(23)	119.541	H(31)-C(10)-C(11)	119.360
H(37)-C(22)-C(21)	120.445	H(31)-C(10)-C(9)	120.225
C(23)-C(22)-C(21)	120.013	C(11)-C(10)-C(9)	120.415
O(24)-C(21)-C(22)	123.222	H(30)-C(9)-C(10)	120.357
O(24)-C(21)-C(20)	116.876	H(30)-C(9)-C(8)	120.699
C(22)-C(21)-C(20)	119.894	C(10)-C(9)-C(8)	118.938
H(36)-C(20)-C(21)	119.188	H(29)-C(8)-C(9)	119.975
H(36)-C(20)-C(19)	120.539	H(29)-C(8)-C(7)	119.28
C(21)-C(20)-C(19)	120.271	C(9)-C(8)-C(7)	120.745
H(35)-C(19)-C(20)	120.711	H(28)-C(7)-C(8)	120.121
H(35)-C(19)-C(18)	119.525	H(28)-C(7)-C(6)	119.078
C(20)-C(19)-C(18)	119.764	C(8)-C(7)-C(6)	120.801
C(23)-C(18)-C(19)	120.199	C(11)-C(6)-C(7)	117.841
C(23)-C(18)-N(17)	119.165	C(11)-C(6)-C(1)	120.880
C(19)-C(18)-N(17)	120.631	C(7)-C(6)-C(1)	121.279
H(34)-N(17)-C(18)	118.625	C(4)-N(5)-C(1)	110.295
H(34)-N(17)-N(16)	121.079	N(12)-C(4)-N(5)	121.775
C(18)-N(17)-N(16)	120.262	N(12)-C(4)-S(3)	122.033
N(17)-N(16)-C(15)	122.150	N(5)-C(4)-S(3)	116.191
C(25)-C(15)-N(16)	121.833	C(4)-S(3)-C(2)	87.819
C(25)-C(15)-C(13)	123.389	H(27)-C(2)-S(3)	119.409
N(16)-C(15)-C(13)	114.772	H(27)-C(2)-C(1)	128.839
C(15)-C(13)-O(14)	122.104	S(3)-C(2)-C(1)	111.751
C(15)-C(13)-N(12)	116.107	C(6)-C(1)-N(5)	120.124
O(14)-C(13)-N(12)	121.788	C(6)-C(1)-C(2)	125.932
H(33)-N(12)-C(13)	120.948	N(5)-C(1)-C(2)	113.944

Table S19: Selected bond lengths (Å) of compound 8e using DFT-method from DMOL3 calculations.

Bond	Length(Å)	Bond	Length(Å)	Bond	Length(Å)
C(29)-N(30)	1.178	C(19)-H(39)	1.091	C(9)-C(10)	1.402
C(28)-H(47)	1.100	C(19)-C(20)	1.392	C(8)-H(33)	1.093
C(28)-H(46)	1.101	C(23)-C(18)	1.409	C(8)-C(9)	1.402
C(28)-H(45)	1.098	C(18)-C(19)	1.411	C(7)-H(32)	1.090
C(27)-H(44)	1.101	N(17)-H(38)	1.027	C(7)-C(8)	1.401
C(27)-H(43)	1.099	N(17)-C(18)	1.410	C(11)-C(6)	1.411
C(27)-C(28)	1.519	N(16)-N(17)	1.317	C(6)-C(7)	1.411
O(25)-C(27)	1.468	C(15)-C(29)	1.430	C(4)-N(12)	1.394
C(24)-O(26)	1.232	C(15)-N(16)	1.323	N(5)-C(4)	1.314
C(24)-O(25)	1.372	C(13)-C(15)	1.500	S(3)-C(4)	1.747
C(23)-H(42)	1.094	C(13)-O(14)	1.236	C(2)-H(31)	1.086
C(22)-H(41)	1.090	N(12)-H(37)	1.018	C(2)-S(3)	1.728
C(22)-C(23)	1.393	N(12)-C(13)	1.389	C(1)-C(6)	1.479
C(21)-C(24)	1.496	C(11)-H(36)	1.092	N(5)-C(1)	1.390
C(21)-C(22)	1.412	C(10)-H(35)	1.093	C(1)-C(2)	1.386
C(20)-H(40)	1.091	C(10)-C(11)	1.400	C(20)-C(21)	1.410
C(9)-H(34)	1.092				

Table S20: Selected bond angles (°) of compound 8e using DFT-method from DMOL3 calculations.

Angle	Degree(°)	Angle	Degree(°)
N(30)-C(29)-C(15)	175.709	C(29)-C(15)-N(16)	120.887
H(47)-C(28)-H(46)	107.883	C(29)-C(15)-C(13)	121.736
H(47)-C(28)-H(45)	108.27	N(16)-C(15)-C(13)	117.353
H(47)-C(28)-C(27)	109.592	C(15)-C(13)-O(14)	123.326
H(46)-C(28)-H(45)	108.626	C(15)-C(13)-N(12)	113.692
H(46)-C(28)-C(27)	111.11	O(14)-C(13)-N(12)	122.972
H(45)-C(28)-C(27)	111.255	H(37)-N(12)-C(13)	120.392
H(44)-C(27)-H(43)	107.82	H(37)-N(12)-C(4)	113.877
H(44)-C(27)-C(28)	111.994	C(13)-N(12)-C(4)	125.723
H(44)-C(27)-O(25)	108.504	H(36)-C(11)-C(10)	118.948
H(43)-C(27)-C(28)	112.216	H(36)-C(11)-C(6)	120.054
H(43)-C(27)-O(25)	108.281	C(10)-C(11)-C(6)	120.985
C(28)-C(27)-O(25)	107.909	H(35)-C(10)-C(11)	119.428
C(27)-O(25)-C(24)	114.86	H(35)-C(10)-C(9)	120.469
O(26)-C(24)-O(25)	123.909	C(11)-C(10)-C(9)	120.099
O(26)-C(24)-C(21)	124.091	H(34)-C(9)-C(10)	120.089
O(25)-C(24)-C(21)	111.991	H(34)-C(9)-C(8)	120.387
H(42)-C(23)-C(22)	120.265	C(10)-C(9)-C(8)	119.51
H(42)-C(23)-C(18)	119.815	H(33)-C(8)-C(9)	119.923
C(22)-C(23)-C(18)	119.92	H(33)-C(8)-C(7)	119.638
H(41)-C(22)-C(23)	119.665	C(9)-C(8)-C(7)	120.439
H(41)-C(22)-C(21)	120.288	H(32)-C(7)-C(8)	120.424
C(23)-C(22)-C(21)	120.041	H(32)-C(7)-C(6)	118.961
C(24)-C(21)-C(22)	123.332	C(8)-C(7)-C(6)	120.610
C(24)-C(21)-C(20)	117.339	C(11)-C(6)-C(7)	118.354
C(22)-C(21)-C(20)	119.329	C(11)-C(6)-C(1)	121.488
H(40)-C(20)-C(21)	118.214	C(7)-C(6)-C(1)	120.154
H(40)-C(20)-C(19)	120.526	C(4)-N(5)-C(1)	110.323
C(21)-C(20)-C(19)	121.259	N(12)-C(4)-N(5)	120.428
H(39)-C(19)-C(20)	121.230	N(12)-C(4)-S(3)	123.264
H(39)-C(19)-C(18)	120.004	N(5)-C(4)-S(3)	116.294
C(20)-C(19)-C(18)	118.740	C(4)-S(3)-C(2)	87.751
C(23)-C(18)-C(19)	120.700	H(31)-C(2)-S(3)	119.605
C(23)-C(18)-N(17)	117.322	H(31)-C(2)-C(1)	128.672
C(19)-C(18)-N(17)	121.977	S(3)-C(2)-C(1)	111.716
H(38)-N(17)-C(18)	117.485	C(6)-C(1)-N(5)	119.390

H(38)-N(17)-N(16)	120.139	C(6)-C(1)-C(2)	126.694
C(18)-N(17)-N(16)	122.370	N(5)-C(1)-C(2)	113.912
N(17)-N(16)-C(15)	119.063		
